# Platelet Cyclic GMP Levels Are Reduced in Patients with Primary Aldosteronism

**DOI:** 10.3390/jcm12227081

**Published:** 2023-11-14

**Authors:** Carla Sala, Marta Rescaldani, Elisa Gherbesi, Gianni Bolla, Cesare Cuspidi, Massimiliano Ruscica, Stefano Carugo

**Affiliations:** 1 Department of Cardio-Thoracic-Vascular Diseases, Foundation IRCCS Ca’ Granda Ospedale Maggiore Policlinico, 20122 Milan, Italygianni.bolla@unimi.it (G.B.); stefano.carugo@unimi.it (S.C.); 2 Department of Clinical Sciences and Community Health, University of Milan, 20122 Milan, Italy; 3 Cardiovascular Department, Association Socio Sanitary Territorial Santi Paolo e Carlo, 20153 Milan, Italy; marta.rescaldani@asst-santipaolocarlo.it; 4 Department of Medicine and Surgery, University of Milano-Bicocca, 20126 Milan, Italy; cesare.cuspidi@unimib.it; 5 Department of Pharmacological and Biomolecular Sciences “Rodolfo Paoletti”, University of Milan, 20122 Milan, Italy

**Keywords:** cyclic guanosine-3′,5′-monophosphate, essential hypertension, nitric oxide, primary aldosteronism

## Abstract

Background and aim: Nitric oxide inhibits platelet aggregation by increasing the second messenger cyclic guanosine-3′,5′-monophosphate (cGMP) through the activation of soluble guanylyl cyclase in target cells. Within this context, the oxidative stress associated with the aldosterone excess impairs the nitric oxide availability. Thus, the aim of the present study was to assess the impact of chronic aldosterone excess on the platelet nitric oxide/cGMP pathway in humans. Methods: The levels of cGMP were evaluated in platelets of male patients, 12 with primary aldosteronism (PA) and 32 with uncomplicated essential hypertension (EH), matched for age and blood pressure (BP) values. Results: PA and EH patients were 52.8 ± 3 years old and 51.6 ± 1.6 years old, respectively. Systolic and diastolic BP were 158 ± 5.0 mmHg and 105.9 ± 2.3 mmHg in PA and did not differ compared to EH patients (156.6 ± 2.4 mmHg and 104.7 ± 1.2 mmHg). Mean aldosterone levels were significantly higher in PA (25.5 ± 8.8 ng/dL) compared toEH (8.11 ± 0.73 ng/dL), whereas potassium was significantly lower in PA (3.52 ± 0.18 mEq/L) compared to EH (4.08 ± 0.04 mEq/L). Aldosterone and potassium were inversely related (r = −0.49, *p* = 0.0006) in the whole study population (*n* = 44). Platelet cGMP was significantly lower in PA (5.1 ± 0.36 pM/10^9^ cells) than in EH (7.1 ± 0.53 pM/10^9^ cells), and in the entire study cohort, it was directly related to plasma potassium (r = 0.43, *p* = 0.0321). Conclusions: These results show an impairment of nitric oxide/cGMP signaling in platelets of PA patients. This effect is likely related to the potassium-depleting effect of chronic aldosterone excess. Future studies are needed to understand whether the platelet nitric oxide/cGMP system is involved in the atherothrombotic events in these patients.

## 1. Introduction

Aldosterone excess exerts multiple adverse cardiovascular effects [1]. In human hypertension, the autonomous hypersecretion of aldosterone by adrenal glomerulosa, independent of the angiotensin II control (i.e., primary aldosteronism (PA)), is responsible forthe most frequent form of secondary hypertension. This condition is characterized by sodium–water retention, volume expansion, and potassium depletion. Besides the effects at the renal tubular level, aldosterone is responsible forcardiac and vascular injury by mechanisms that involve a variety of molecular messengers with proinflammatory and profibrotic actions and the production of reactive oxygen species [2]. In endothelial cells, aldosterone can induce endothelial dysfunction via multiple pathways (e.g., by reducing the bioavailability of the vasodilator nitric oxide and by increasing the synthesis of the vasoconstrictor endothelin) [3].

Nitric oxide regulates multiple physiological processes, including vascular tone and platelet function, by activating soluble guanylyl cyclaseand the synthesis of intracellular cyclic guanosine-3′,5′-monophosphate (cGMP) [4]. A defect of nitric oxide/cGMP signaling and oxidative stress is a hallmark of endothelial dysfunction-promoting vasoconstriction, inflammation, and thrombosis associated with major cardiovascular risk factors [4]. On this matter, in human endothelial cells, aldosterone was able to inhibit the activity of endothelial nitric oxidesynthase and the production of cGMP, an effect thatwas reverted by the mineralocorticoid receptor antagonist, eplerenone [5]. In pre-clinical models, aldosterone infusion impaired endothelium-dependent relaxation, an effect thatwas associated with increased oxidative stress in the vascular wall [2]. Finally, in the clinical setting, chronic aldosterone excess in patients with PA impairs flow-mediated arterial vasodilation, a surrogate marker of endothelial function largely mediated by nitric oxide availability, an effect that was partly reverted by mineralocorticoid receptors blockade [6].

Within this context, the components of the nitric oxide/cGMP pathway, including nitric oxide synthase and soluble guanylyl cyclase, were identified in platelets cytoplasm since the 80s [7]. The nitric oxide/cGMP signaling is the major pathway involved in the inhibition of platelet adhesion and aggregation [8,9]. In mice knock-out for nitric oxide-sensitive guanylyl cyclase, the marked increase of blood pressure (BP) was associated with the inability of nitric oxide to inhibit platelet aggregation in response to aggregating agents, at difference from platelets from wild-type animals [10].

Since no data are available about the effects of chronic aldosterone excess on the platelet nitric oxide/cGMP system in humans, we compared cGMP levels in platelets of patients with PA with a group of patients with essential hypertension (EH), matched for age, gender and BP values.

## 2. Materials and Methods

### 2.1. Subjects

Twelve Caucasian patients with PA and 32 with uncomplicated EH (all males) matched for age and clinic BP values were included in this retrospective, cross-sectional, single-centerstudy. The diagnosis of PA in patients with resistant hypertension and spontaneous or diuretic-induced hypokalemia was based on plasma aldosterone levels ≥ 15 ng/dL, low plasma renin activity (PRA), aldosterone-to-renin ratio (ARR) ≥ 40 ng/dL per ng mL^1^ h^1^ and plasma aldosterone > 10 ng/dL after intravenous saline load (0.9% sodium chloride 2 L/4 h); this latter as a confirmatory test [11]. Morphofunctional studies (computed tomography and ^131^I adrenocortical scintigraphy after dexamethasone administration) identified bilateral hyperplasia in ninepatients and monolateral adenoma in threecases. Office BP was measured usingan automated device (Omron M6 Comfort) and reported as the average values of threemeasurements taken at 1 min intervals after the subject was resting for 5 min in the sitting position. The majority of patients wereout of any antihypertensive treatment since these individuals were hypertensive atfirst diagnosis at the time of the recruitment. In the remaining patients, any antihypertensive treatment interfering with the renin-angiotensin-system (i.e., angiotensin-converting enzyme inhibitors, sartans, β-blockers, and diuretic) was withdrawn for 2 weeks before blood sampling. However, when BP was >160/110 mmHg (3 PA/4 EH), a calcium channel-blocker verapamil and/or the alpha-blocker doxazosine were administered. No other drugs, including mineralocorticoid receptor antagonists, statins, or antiplatelet drugs, were taken at the time of the study. BMI was calculated as weight/height (kg/m^2^). Informed consent was obtained from all subjects.

### 2.2. Blood Sampling

Blood for routine chemistry and humoral determinations was sampled from an antecubital vein after the subjects were resting supine for 60 min, between 9 and 11 a.m. Blood was immediately processed for platelets separation and cGMP extraction; plasma for humoral determinations, renin activity PRA, aldosterone, atrial natriuretic peptide, and plasma cGMP was separated and stored at −80 °C until assay.

### 2.3. Assays

Platelet cGMP was measured usingradioimmunoassay (Amersham-GR Healthcare, Amersham, UK) on acid extracts of washed platelets, as previously reported [12]. Briefly, peripheral venous blood (13 mL) was collected in 5 mmol/L ethylenediaminetetraacetic acid, 10 U/mL heparin, 3.8% sodium citrate, and 0.55 mmol/L isobutyl methylxanthine. Then, blood was centrifuged at 200× *g* for 20 min at 20 °C to obtain platelet-rich plasma. The platelet-rich plasma was centrifuged at 1000× *g* for 10 min at 20 °C, the supernatant discarded, and the pellet suspended in 550 µL of modified Tyrode’s solution (5 mmol/L HEPES and 0.35% bovine serum albumin, pH 7.4). A 50-µL aliquot was used for manual platelet count. The remaining suspension was acid-precipitated with trichloroacetic acid (final concentration 6%) and centrifuged at 2000× *g* for 15 min at 4 °C. The pellet was discarded, and the supernatant was washed with glacial ether (5×) to extract trichloroacetic acid. Samples were frozen at −80 °C until assayed. cGMP was assayed usingradioimmunoassay with a commercial kit (Amersham-GR Healthcare, Amersham, UK). The intra- and inter-assay coefficients of variation were <10 and <15%, respectively.

To set this methodology in our laboratory, platelet cGMP was previously measured in 28 healthy normotensive individuals (Supplementary Table S1).

PRA and aldosterone were measured usingradioimmunoassay with commercial kits (Technogenetics, Milan, Italy). Plasma atrial natriuretic peptide (ANP) and cGMP were measured usingradioimmunoassay (Amersham-GR Healthcare, UK). Plasma electrolytes, sodium and potassium, and creatinine were measured usingstandardized automated methods. The glomerular filtration rate (eGFR) was calculated usingthe EPI-CKD formula.

Plasma lipids (total cholesterol, high-density lipoprotein cholesterol (HDL-C), and triglycerides) were measured usingcertified enzymatic techniques on a Roche c311 autoanalyzer. Low-density lipoprotein cholesterol (LDL-C) was calculated usingthe Friedewald equation: total cholesterol minus HDL-C minus triglycerides/5. Non-HDL-C was calculated as total cholesterol minus HDL-C.

### 2.4. Statistical Analysis

Data are expressed as mean ± sem; logarithmic transformation was applied for plasma aldosterone displaying a skewed distribution. Univariate associations between continuous variables were determined usingPearson’s correlation. Differences between the two groups were assessed using anunpaired Student’s *t*-test. A correlation between variables was evaluated usinglinear regression analysis. Statistical significance was set for *p*-value ≤ 0.05. Analyses were performed usingPrism version 8.0 (GraphPad, Boston, MA, USA). Assuming a mean standard deviation within each group of 2.12 (3.0 in EH and 1.2 in PA), we obtained an effect size d of 0.94 and α error probability of 0.03 and a 1-β error probability of 0.70 (70%) (Package G*Power). 

## 3. Results

### 3.1. Clinical Characteristics of Patients

As shown in Table 1, age, BMI, BP, glycemia, lipids/lipoproteins (total cholesterol, LDL-C, HDL-C, non-HDL-C, and TG), and renal function did not differ between PA and EH patients. Plasma potassium was significantly lower in PA (3.52 ± 0.18 mEq/L; range 2.2–4.3 mEq/L) vs. EH (4.08 ± 0.04 mEq/L; range 3.5–4.6 mEq/L), whereas sodium levels were increased in PA (143.8 ± 1.2 mEq/L; range 138–153 mEq/L) vs. EH (141.8 ± 0.4 mEq/L; range 137–148 mEq/L). Further, potassium was below the normal range (3.5 mEq/L) in sixPA and in none of the EH patients; sodium was above the normal range (145 mEq/L) in fivePA and in twoEH patients. Relative to humoral parameters, PRA was suppressed, and aldosterone was almost three-fold higher in PA (25.5 ± 8.7 ng/dL) compared to EH (8.11 ± 0.73 ng/dL). The aldosterone-to-renin ratio (230 ± 90) was more than five-fold higher than the 40-cut-off limit for the diagnosis of PA [13]. Plasma ANP tended to be higher in PA than EH; no between-group difference was found in plasma cGMP levels.

**Figure 1:**
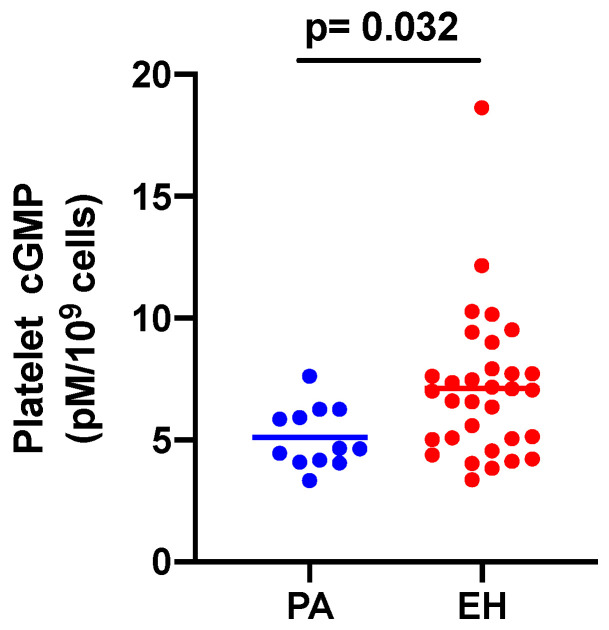
Scatter-plot of cGMP in platelets. Primary aldosteronism (PA, *n* = 12; blue dots) and Essential hypertension (EH, *n* = 32; red dots); *p*, values. cGMP, cyclic guanosine-3′,5′-monophosphate.

Differently from plasma cGMP (Table 1), the levels of cGMP in platelets were lower in PA patients compared to EH (5.1 ± 0.36 vs. 7.1 ± 0.53 pM/10^9^ cells, *p* = 0.0321; Figure 1).

### 3.2. Correlation Analyses

**Figure 2:**
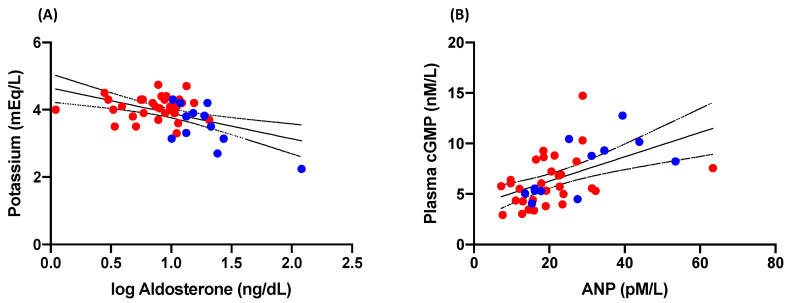
Correlation analyses between (**A**) plasma levels of potassium and aldosterone and (**B**) between plasma levels of cGMP and ANP in patients with primary aldosteronism (*n* = 12; blue dots) and essential hypertension (*n* = 32; red dots). cGMP, cyclic guanosine-3′,5′-monophosphate; ANP, atrial natriuretic peptide.

In the whole cohort, an inverse correlation was found between aldosterone and potassium (r = −0.49, *p* = 0.0006; Figure 2A). Conversely, a significant positive relationship between plasma levels of ANP and cGMP was present (r = 0.56, *p* < 0.0001; Figure 2B).

**Figure 3:**
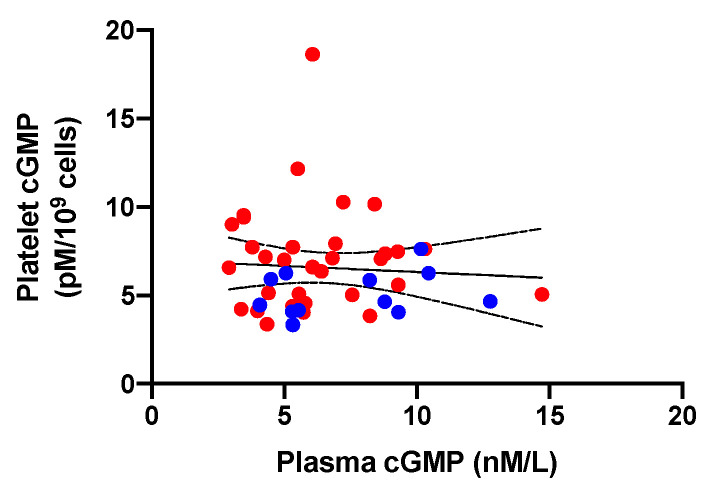
Correlation analysis between platelet and plasma cGMP in patients with primary aldosteronism (*n* = 12; blue dots) and essential hypertension (*n* = 32; red dots). cGMP, cyclic guanosine-3′,5′-monophosphate.

The levels of cGMP in platelets and in plasma were not correlated (r = −0.065, *p* = 0.67; Figure 3).

**Figure 4:**
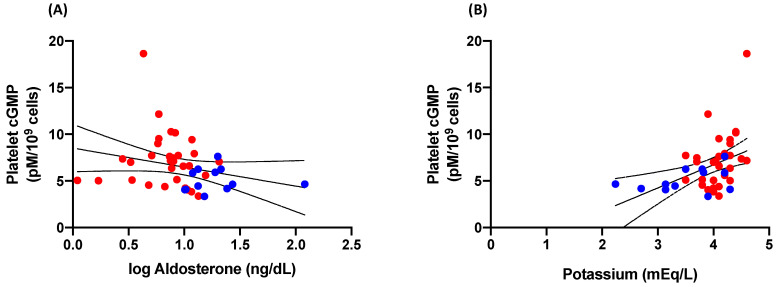
Correlation analyses between platelet cGMP and (**A**) aldosterone and (**B**) potassium in patients with primary aldosteronism (*n* = 12; blue dots) and essential hypertension (*n* = 32; red dots). cGMP, cyclic guanosine-3′,5′-monophosphate.

Finally, platelet cGMP was not related to plasma aldosterone (r = −0.25, *p* = 0.11; Figure 4A), whereas a direct relationship between platelet cGMP and plasma potassium was present (r = 0.43, *p* = 0.004; Figure 4B).

## 4. Discussion

The present study demonstrates that cGMP levels in platelets of patients with PA were lower compared to EH subjects. Considering that cGMP is a marker of nitric oxide activity, this finding is in line with an impaired platelet nitric oxide/cGMP pathway in a clinical condition of chronic aldosterone excess, such as PA, the most frequent form of secondary hypertension.Increments of cGMP from the nitric oxide-activated soluble guanylyl cyclaseare associated withthe anti-aggregatory, anti-thrombotic effects of nitric oxide [9]; thus, a defect of nitric oxide/cGMP signaling is compatible with a pro-thrombotic condition in PA patients. In uncomplicated EH, our group previously documented that lower cGMP levels inplatelets were related to an enhanced aggregating response to Epinephrine [12].

In anucleated platelets, the possibility of nongenomic actions of aldosterone, mediated by the production of reactive oxygen species, should be considered. In addition to renal tubular cells, mineralocorticoid receptors mediating genomic and non-genomic effects of aldosterone have been identified in nonepithelial tissues, including endothelial cells, vascular smooth muscle cells, and human platelets [1,14]. The epithelial sodium channel, expressed in the membrane of vascular endothelium and regulated by aldosterone through mineralocorticoid receptors, stimulates Na^+^ current, leading to an impairment of endothelial nitric oxide synthase and reduced nitric oxide production [3]. In vascular smooth muscle cells exposed to aldosterone (10^−7^ M), oxidation of soluble guanylyl cyclase and its desensitization to nitric oxide resulted in reduced levels of cGMP [15]. In experimental thrombosis, the acute administration of aldosterone in rats increases platelet aggregates within a few minutes and induces a thrombotic effect that involves non-genomic signaling and is partially mediated by mineralocorticoid receptors [16]. The mineralocorticoid antagonist eplerenone inhibits platelet activation, as assessed by fibrinogen binding in animals with congestive heart failure [17]. In atherosclerotic mice, aldosterone treatment for ninety days enhanced thrombus formation following arterial injury [18].

In the present study, the role of confounding factors associated with endothelial dysfunction and reduced nitric oxide/cGMP activity, such as age, gender, and hypertension, can be excluded, as the two groups of patients were matched for these parameters. The process of aging has been associated with oxidative stress and an impairment of the nitric oxide/cGMP pathway, documented by a progressive decrease inplatelet cGMP in experimental animals [19] and an impaired endothelium-dependent vasodilation in humans [20]. Women benefit from the vasoprotective effects of estrogens, which cause long-term upregulation of eNOS expression with an increased nitric oxide availability [21].

Arterial hypertension is associated with oxidative stress, reduced nitric oxide bioavailability, and impaired endothelium-dependent vasodilation documented in the coronary and peripheral circulation of hypertensive subjects compared to normotensive counterparts [22].

The question of whether potassium depletion elicited by aldosterone and the associated hypokalemia may be responsible for some of the adverse effects of aldosterone on the cardiovascular system is still debated. Experimental and clinical evidence supports the beneficial effects of potassium supplements on the cardiovascular system and the detrimental role of aldosterone excess in accelerating target-organ damage [23].

In vitro studies indicate that increasing extracellular concentrations of K^+^ from 4 to 8 mM causes a swelling of endothelial cells, decreased cell stiffness, and increased nitric oxide release, as assessed by nitrite concentration in the supernatant. This effect is blunted by the presence of 0.45 nM aldosterone in a high-sodium medium [24]. Since the 80s, Tobian’s group documented the beneficial effects of potassium supplements to prevent aortic intimal lesions in spontaneously hypertensive rats [25], to improve endothelium-dependent relaxation as well as to enhance endothelial nitric oxide production in carotid arteries of Dahl rats [26]. In a clinical setting, higher dietary potassium intake improves flow-mediated dilation as well as arterial compliance in hypertensive patients [23] and reduces the risk of stroke and cardiovascular disease, as documented in a meta-analysis of prospective studies [27].

Our observation that platelet cGMP levels were more significantly related to potassium decrements than to aldosterone increments supports the view that potassium depletion induced by aldosterone may be the final mediator of the impairment of platelet nitric oxide-cGMP pathway in PA patients. Concerning plasma cGMP, which was unrelated to platelet cGMP, this finding supports the existence of two different pools of the second messenger cGMP: (a) an intracellular pool derived from the nitric oxide-induced activation of soluble guanylyl cyclase in platelets cytoplasm, and (b) a circulating pool derived from the activation of particulate guanylyl cyclase, the biologically active receptors of natriuretic peptides, localized on endothelial cells [28] and not on platelets membrane [29]. The direct significant relationship between circulating levels of ANP in a condition of volume expansion, such as PA and plasma cGMP, may support this view.

Considering that reduced activity of the platelet nitric oxide/cGMP system is associated withenhanced adhesion/aggregation in experimental [8] and clinical settings [4], our data in patients with PA support the hypothesis that the impaired nitric oxide/cGMP signaling and the enhanced platelet activity may contribute to the higher prevalence of atherothrombotic events in this clinical condition [30].

The results of the present study have to be interpreted within the context of potential limitations. First, the study was limited to the measurement of platelets cGMP. Moreover, responses to exogenous aggregating agents were not tested. Second, different degrees of platelet activation in all patients can not be excluded as markers of platelet activation, such as P-selectin on the membrane surface, were not assessed, along with circulating thrombosis biomarkers. Furthermore, we did not assess the effect of mineralocorticoid receptor antagonists on platelets cGMP in patients with primary aldosteronism. Third, the numerosity of the PA group was limited since we recruited highly selected individuals (e.g., none werepreviously treated with mineral receptor antagonists). Fourth, our observations refer to the male gender, as females were excluded from our study in order to avoid confounding effects given by estrogens on the nitric oxide/cGMP system. Fifth, among the components of the natriuretic peptides system, we evaluated the levels of ANP and not those of brain natriuretic peptides.

## 5. Conclusions

Data fromthe present study support therole of aldosterone excess as a negative modulator of platelet nitric oxide/cGMP activity in humans, an effect that may be mediated by hypokalemia induced by aldosterone. Thus, it could be speculated that the impairment of nitric oxide/cGMP signaling in platelets of patients with PA may enhance the response to the aggregating stimuli in this clinical condition. The favorable effects of pharmacological interventions on this pro-thrombotic state remain to be determined.

## Figures and Tables

**Table 1:** Clinical characteristics in male patients with primary aldosteronism and essential hypertension.

Baseline Characteristics	PA (*n* = 12)	EH (*n* = 32)	*p*-Value
Age, years	52.8 ± 3.0	51.6 ± 1.6	0.779
BMI, kg/m^2^	28.7 ± 1.2	27.6 ± 1.2	0.437
Systolic BP, mmHg	158.3 ± 5.0	156.6 ± 2.4	0.74
Diastolic BP, mmHg	105.9 ± 2.3	104.7 ± 1.2	0.61
Heart rate, beats/min	74.5 ± 3.6	73.0 ± 2.0	0.70
Glycemia, mg/dL	93.4 ± 5.6	93.8 ± 2.5	0.94
TC, mg/dL	210.9 ± 13.4	208.4 ± 6.71	0.87
LDL-C, mg/dL	134.7 ± 15.5	137.1 ± 4.27	0.83
HDL-C, mg/dL	41.9 ± 3.68	42.9 ± 2.10	0.83
non-HDL-C, mg/dL	169.2 ± 17.2	166.5 ± 5.8	0.81
TG, mg/dL	158.7 ± 31.3	144.6 ± 15.4	0.70
eGFR, mL/min	81.3 ± 7.7	81.6 ± 2.3	0.96
Sodium, mEq/L	143.8 ± 1.2	141.8 ± 0.4	**0.048**
Potassium, mEq/L	3.52 ± 0.18	4.08 ± 0.04	**0.0002**
PRA, ng/mL/h	0.13 ± 0.01	0.42 ± 0.09	**0.05**
Aldosterone, ng/dL	25.5 ± 8.7	8.11 ± 0.73	**0.0024**
ARR	230 ± 89.9	30.9 ± 3.7	**0.0007**
ANP, pM/L	27.9 ± 3.7	20.6 ± 1.8	**0.063**
plasma cGMP, nM/L	7.45 ± 0.82	6.24 ± 0.45	0.184

ARR, aldosterone-to-renin ratio; ANP, atrial natriuretic peptide; BMI, body mass index; BP, blood pressure; eGFR, estimated glomerular filtration rate; EH, essential hypertension; HDL-C, high-density lipoprotein cholesterol; LDL-C, low-density lipoprotein cholesterol; PA, primary aldosteronism; PRA, renin activity; TC, total cholesterol; TG, triglycerides. Data are presented as mean ± sem. Differences have been established using an unpaired Student’s *t*-test. Significant between-group differences are highlighted in bold.

## Data Availability

Data will be available uponrequest at the corresponding authors’ addresses.

## Supplementary Materials

The following supporting information can be downloaded at https://www.mdpi.com/article/10.3390/jcm12227081/s1, Table S1: Clinical and humoral characteristics in 28 normotensive subjects.
