# Supplementary material for: Platelet Cyclic GMP Levels Are Reduced in Patients with Primary Aldosteronism
Source: J Clin Med. 2023 Nov 14;12(22):7081. doi: 10.3390/jcm12227081 (PMC10672647; doi:10.3390/jcm12227081)
Supplement: Supplementary file 1 [file jcm-12-07081-s001.zip › jcm-2599417-supplementary.pdf]

Table S1. Clinical and humoral characteristics in 28 normotensive subjects.

	Parameters	Range
**Sex (M/F)**	25/3	
**Age**, years	39.9 ± 2.8	22 - 69
**BMI,** kg/m^2	25.0 ± 0.6	19 - 33
**Systolic BP**, mmHg	118.8 ± 2.3	95 - 140
**Diastolic BP**, mmHg	77.3 ± 1.1	65 - 85
**Heart rate**, beats/min	66.2 ± 1.6	51 - 81
**Creatinine**, mg/dL	1.0 ± 0.1	0.7 - 1.3
**Glucose,** mg/dL	87.4 ± 2.4	70 - 104
**Sodium**, mEq/L	142.3 ± 0.3	142 - 143
**Potassium**, mEq/L	4.43 ± 0.12	4.2 - 4.6
**TC,** mg/dL	190.2 ± 8.4	146 - 260
**LDL-C,** mg/dL	172.0 ± 8.9	135 - 247
**HDL,** mg/dL	47.0 ± 2.8	31 - 71
**TG,** mg/dL	97.3 ± 12.2	50 - 229
**platelet cGMP, pM/10^9 cells**	6.79 ± 0.48	3.62 - 15.2
**ANP, pM/L**	17.4 ± 1.4	7.4 - 28.6
**plasma cGMP, nM/L**	6.15 ± 0.43	1.7 - 10.4

BMI, body mass index; BP, blood pressure; HDL-C, high-density lipoprotein cholesterol; LDL-C, low-density lipoprotein cholesterol; TC, total cholesterol; TG, triglycerides; cGMP, cyclic guanosine-3′,5′-monophosphate; ANP, atrial natriuretic peptide. Data are presented as mean ± sem.
